# Correction: Li et al. Cherry Polyphenol Extract Ameliorated Dextran Sodium Sulfate-Induced Ulcerative Colitis in Mice by Suppressing Wnt/β-Catenin Signaling Pathway. *Foods* 2022, *11*, 49

**DOI:** 10.3390/foods15091484

**Published:** 2026-04-24

**Authors:** Fuhua Li, Huiming Yan, Ling Jiang, Jichun Zhao, Xiaojuan Lei, Jian Ming

**Affiliations:** 1 College of Food Science, Southwest University, Chongqing 400715, China; fuhualee92@163.com (F.L.); yhm2639722584@163.com (H.Y.); 15823556360@163.com (L.J.); jichunzhao@swu.edu.cn (J.Z.); xjuanlei@swu.edu.cn (X.L.); 2 Research Center of Food Storage & Logistics, Southwest University, Chongqing 400715, China

In the original publication [1], there were mistakes in both Figure 7 and its caption. Specifically, Figure 7D,E contain duplicate content. In the caption of Figure 7, the expressions “ZO-1 (A–E)” and “occludin (a–e)” should be corrected to “ZO-1 (a–e)” and “occludin (A–E)”, respectively. The corrected Figure 7 and its caption are presented below. The authors state that the scientific conclusions are unaffected. This correction was approved by the Academic Editor. The original publication has also been updated.

**Figure 7:**
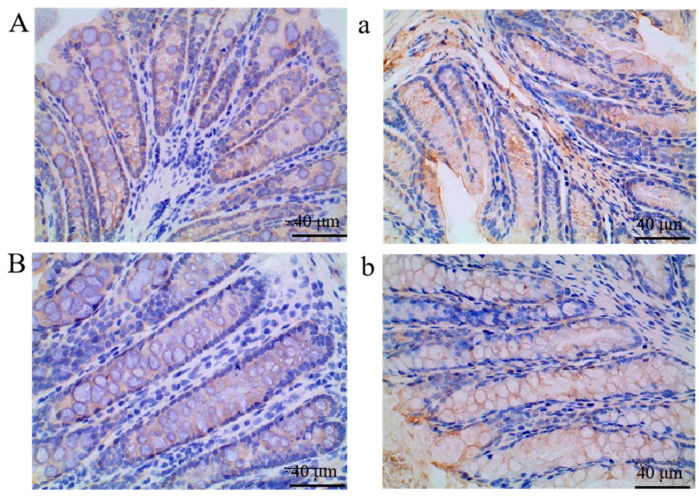

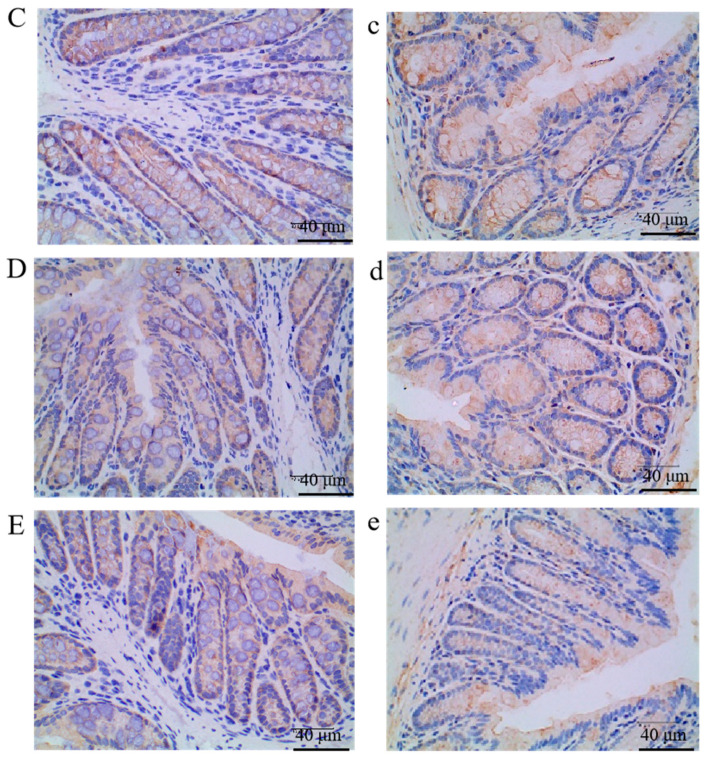
The staining photographs of immunostained analysis of ZO-1 (**a**–**e**) and occludin (**A**–**E**) proteins in colonic tissue of mice (400×). (**A**,**a**), N mice; (**B**,**b**), C mice; (**C**,**c**), P mice; (**D**,**d**),H mice; (**E**,**e**), L mice. Where, N refers the normal group (N); C refers the control group (C); P refers the positive control group (P); H refers the high dose group (H); L refers the low dose group (L).

Apart from the above corrections, there is a correction to Academic Editors’ name. 

The authors state that the scientific conclusions are unaffected. This correction was approved by the Academic Editor. The original publication has also been updated.
