# Supplementary material for: Cherry Polyphenol Extract Ameliorated Dextran Sodium Sulfate-Induced Ulcerative Colitis in Mice by Suppressing Wnt/β-Catenin Signaling Pathway
Source: Foods. 2021 Dec 26;11(1):49. doi: 10.3390/foods11010049 (PMC8750665; doi:10.3390/foods11010049)
Supplement: Supplementary file 1 [file foods-11-00049-s001.zip › foods-1476793-supplementary.pdf]

Supplementary Tables:

Table S1. The polyphenols content and antioxidant capacities in vitro of the free and bound polyphenols extracts

	Content (mg GAE/g DW)	DPPH EC_{50} (μg/mL)	FRAP EC_{50} (μg/mL)	ABTS EC_{50} (μg/mL)	ORAC (μmol TE/g DW)
Free	6.71± 0.01 [a]	32.86±0.15 [b]	65.21±2.51 [b]	35.33±0.22 [a]	297.78±6.69 [a]
Bound	0.13 ± 0.001 [b]	67.28±0.86 [a]	83.91±1.81 [a]	25.08±0.38 [b]	8.00±1.86 [b]

Different superscript letters indicate difference were significant at $p<0.05$ level.

Table S2. The phenolic composition of the free polyphenols extract of cherry identified by HPLC

Phenolic compounds	HPLC-DAD λmax (nm)	*Rt.*(min)	Regression equations (x is the content; y is the peak area)	R^2	Content (μg/g DW)[a]
Chlorogenic acid	320	18.99	y = 36945x - 19198	0.9997	153.81±17.90
Cyanidin 3-*O*-glucoside	520	19.67	y = 29301x - 39175	0.9991	1276.48±99.77
Syringic acid	280	24.02	y = 37908x - 2814.1	0.9989	76.57±2.08
Catechin	280	25.28	y = 18415x - 20862	0.9990	84.93±10.34
Rutin	360	31.55	y = 21379x + 6825.2	0.9985	177.44±18.39

[a] It refers to micrograms of the identified component per gram edible part of cherry in dry weight.

Table S3. The histological scores of colonic tissue of the tested mice

Groups	Score
N	0.00±0.00[a]
C	7.00±0.00[b]
P	1.33±0.58[c]
H	1.33±0.58[c]
L	2.67±0.58[d]

Different superscript letters represent differences were significant at p<0.05 level.

Supplementary Figures:

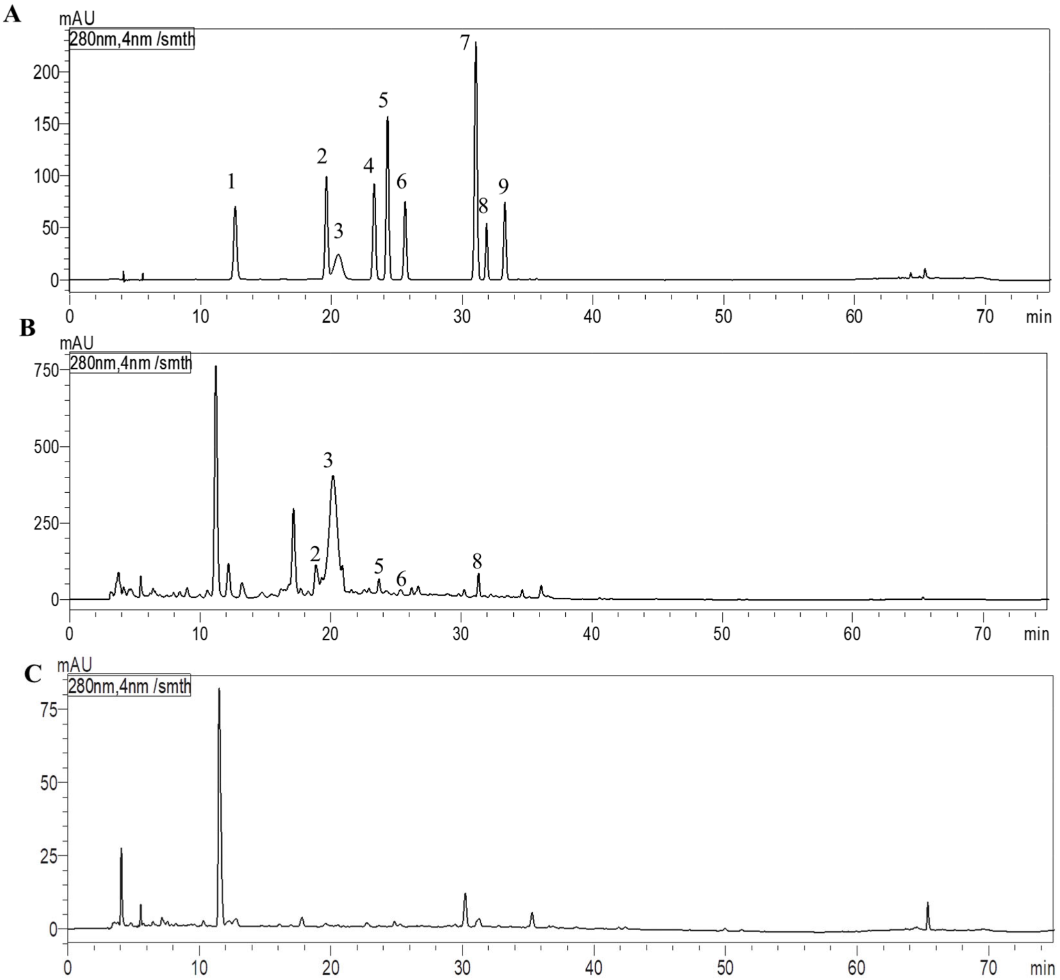

Figure S1. The HPLC chromatograms of phenolic standards (A), phenolic compounds of the free extract (B) and phenolic compounds of the bound extract (C). 1. Protocatechuic acid; 2. Chlorogenic acid; 3. Cyanidin 3-*O*-glucoside; 4. Vanillic acid; 5. Syringic acid; 6. Catechin; 7. *p*-coumaric acid; 8. Rutin; 9. Erucic acid. Phenolic compounds were identified by comparing with both the maximum UV absorption wavelength and the retention time of the phenolic standards.

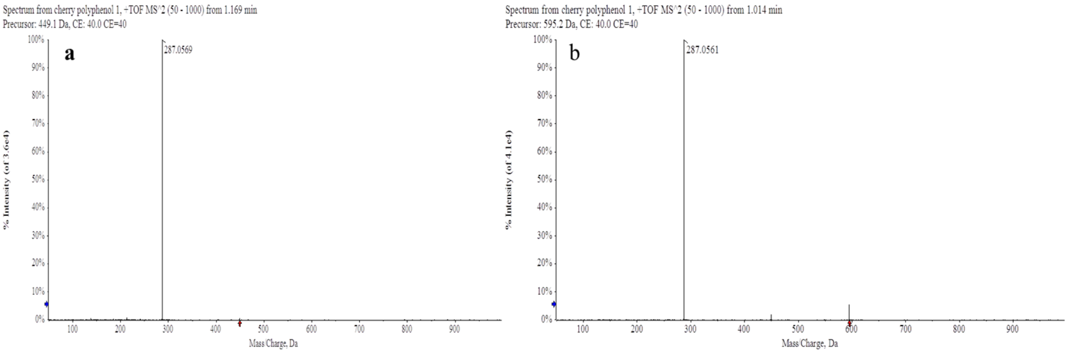

Figure S2. The secondary mass spectrums of cyanidin 3-*O*-glucoside (a) and cyanidin 3-*O*-rutinoside (b)

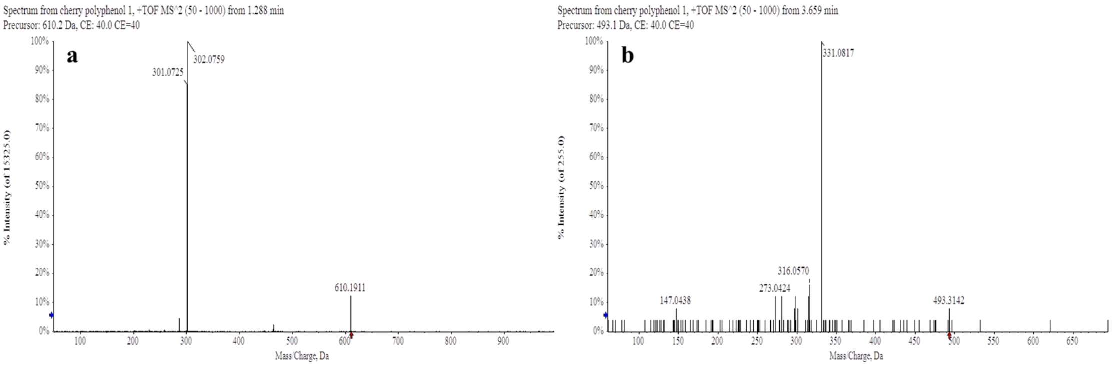

Figure S3. The secondary mass spectrums of peonidin 3-*O*-rutinoside (a) and malvidin-hexoside (b)

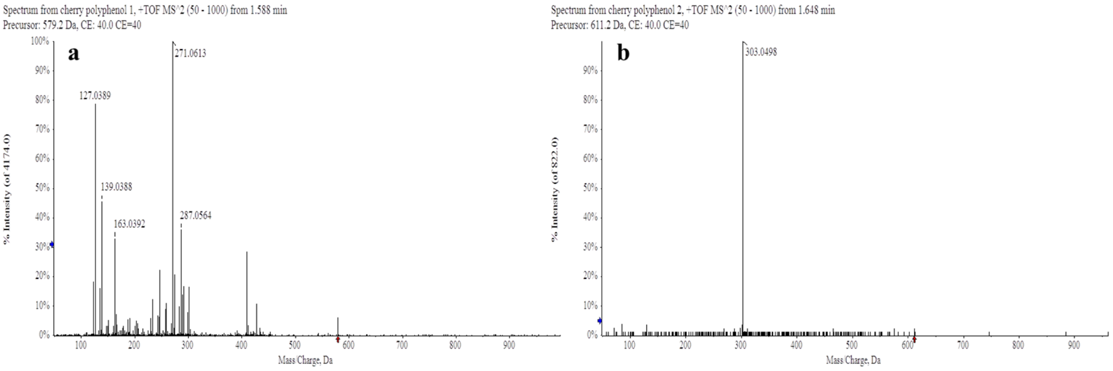

Figure S4. The secondary mass spectrums of pelargonidin 3-rutinoside (a) and delphinidin 3-*O*-rutinoside (b)

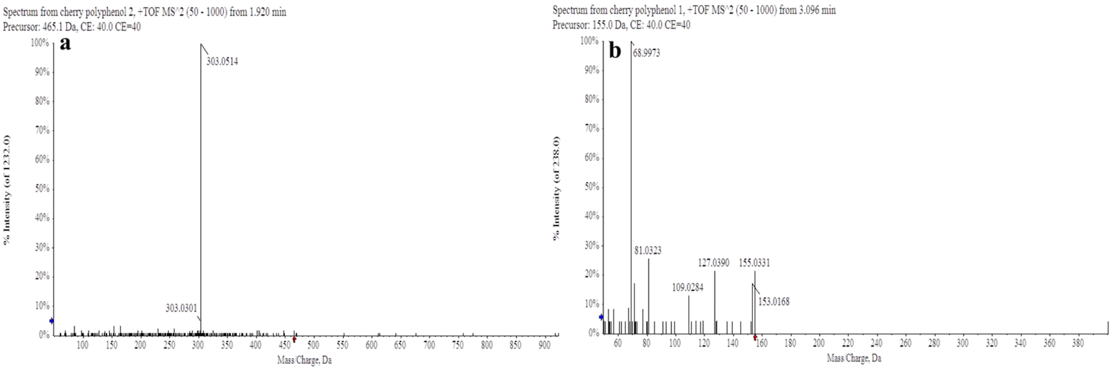

Figure S5. The secondary mass spectrums of delphinidin-hexoside (a) and protocatechuic acid (b)

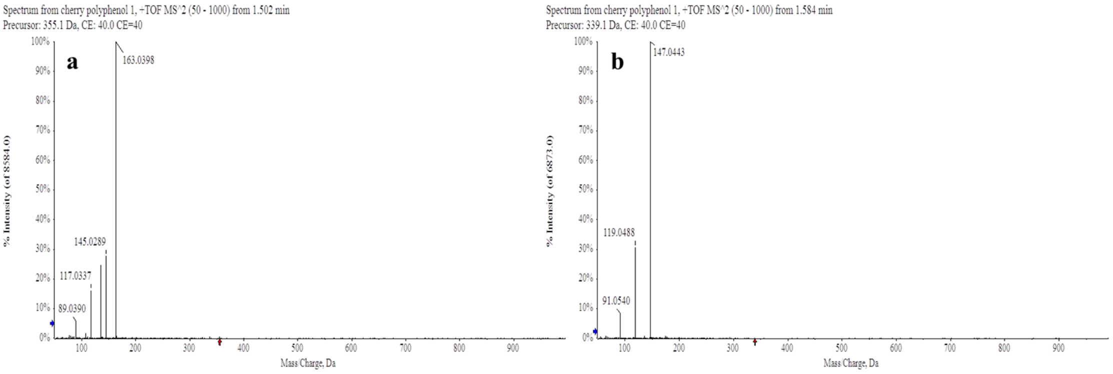

Figure S6. The secondary mass spectrums of caffeoylquinic acid (a) and 3-*p*-coumarylquinic acid (b)

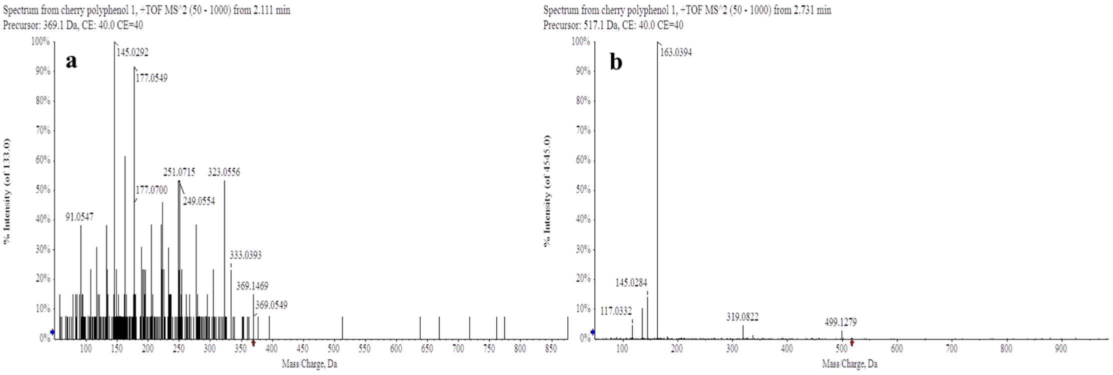

Figure S7. The secondary mass spectrums of feruloylquinic acid (a) and dicaffeoylquinic acid (b)

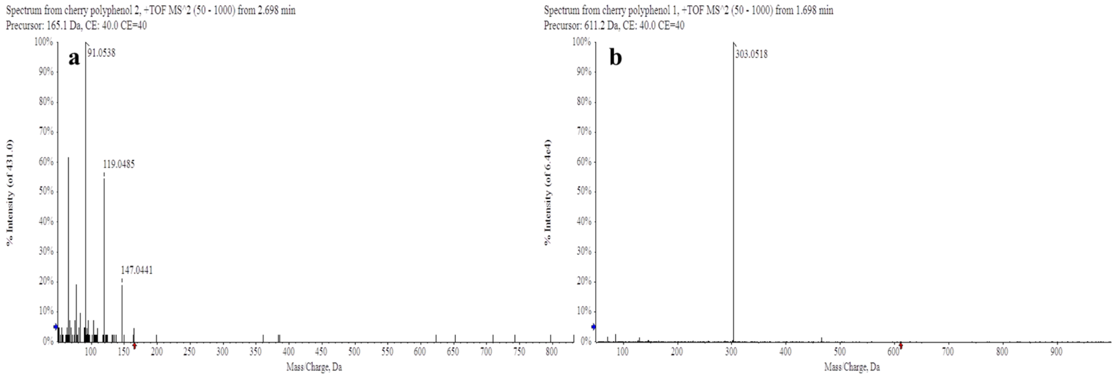

Figure S8. The secondary mass spectrum of *p*-coumaric acid (a) and quercetin 3-*O*-rutinoside (b)

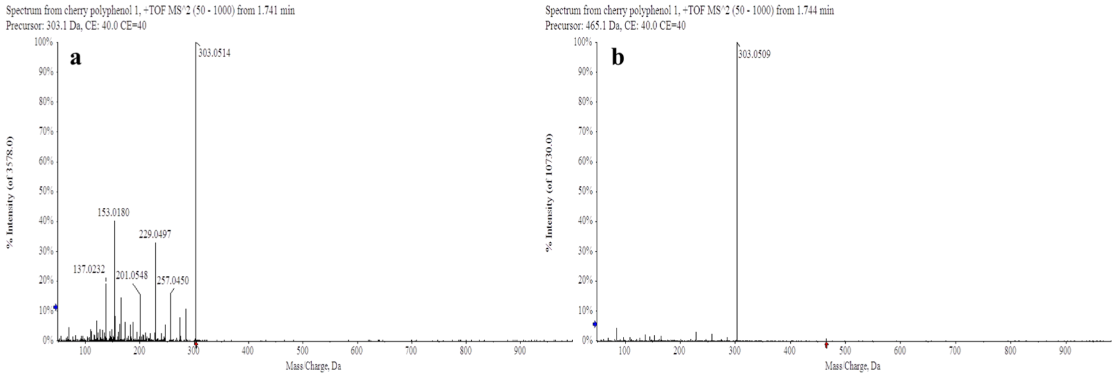

Figure S9. The secondary mass spectrums of quercetin 3-*O*-hexoside (a) and quercetin (b)

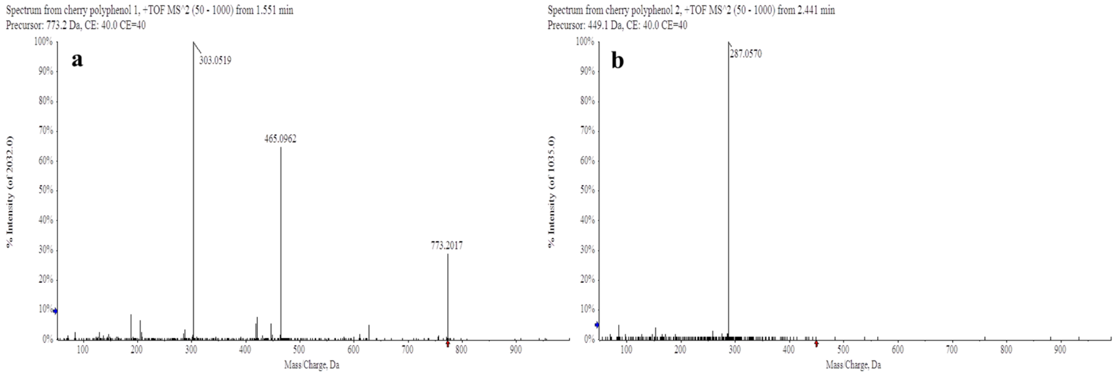

Figure S10. The secondary mass spectrums of quercetin 7-*O*-glucoside-3-*O*-rutinoside (a) and kaempferol 3-glucoside (b)

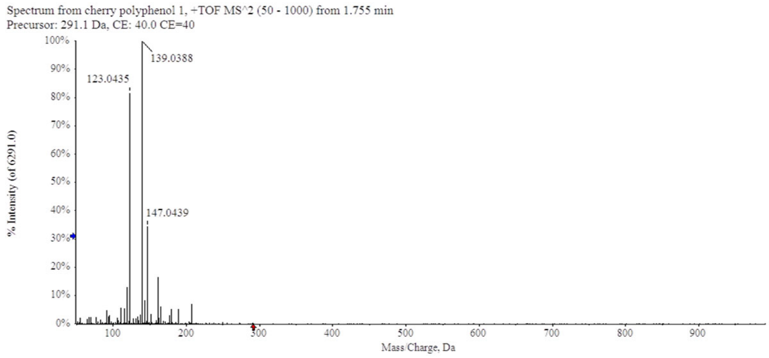

Figure S11. The secondary mass spectrum of catechin
